# Protective effect of *Sophora pachycarpa* seed extract on carbon tetrachloride-induced toxicity in rats

**DOI:** 10.1186/s12906-022-03554-9

**Published:** 2022-03-17

**Authors:** Hamed Aramjoo, Pouria Mohammadparast-Tabas, Tahereh Farkhondeh, Mahmoud Zardast, Marzieh Makhdoumi, Saeed Samarghandian, Zahra Kiani

**Affiliations:** 1 Student Research Committee, Birjand University of Medical Sciences, Birjand, Iran; 2 Medical Toxicology and Drug Abuse Research Center (MTDRC), Birjand University of Medical Sciences, Birjand, Iran; 3 Department of Pharmacology, School of Pharmacy, Birjand University of Medical Sciences, Birjand, Iran; 4 Noncommunicable Diseases Research Center, Neyshabur University of Medical Sciences, Neyshabur, Iran

**Keywords:** *Sophora pachycarpa*, Carbon tetrachloride, Antioxidant, Oxidative stress, Rat

## Abstract

The aim of the present study was to investigate the protective effect of the *Sophora pachycarpa (S. pachycarpa)* seed extract against carbon tetrachloride-induced toxicity on body organs, blood, and biochemical factors. In this investigation, 40 male Wistar rats weighing 200–250 g were randomly divided into 5 groups: group I was used as control, group II received carbon tetrachloride (CCl_4_) (IP, 1 mL/kg) on day 21, group III and group IV received *S. pachycarpa* seed extract at doses of 150 mg/kg and 300 mg/kg, respectively for 21 days by oral gavage and CCl_4_ on day 21, group V received silymarin (300 mg/kg) for 21 days by oral gavage and CCl_4_ on day 21. CCl_4_ showed an increase of serum renal and hepatic markers creatinine, urea, blood urea nitrogen (BUN), and uric acid, alkaline phosphatase (ALP), aspartate aminotransferase (AST), and alanine aminotransferase (ALT). Also, it significantly increased MDA level, and decreased CAT, FRAP, GSH, and SOD in the liver and kidney tissues. These changes and also hematological and histopathological alterations were significantly ameliorated by *S. pachycarpa* seed extract before CCl_4_ administration. In conclusion, the data obtained in our investigation confirm the protective effect of *S. pachycarpa* against acute exposure to CCl_4_-induced organ toxicity in rats.

**Supplementary Information:**

The online version contains supplementary material available at 10.1186/s12906-022-03554-9.

## Introduction

Carbon tetrachloride (CCl_4_) is a toxic, colorless, volatile, and non-flammable liquid. The name of this chlorinated hydrocarbon is tetrachloromethane, according to the International System of Pure and Applied Chemistry (IUPAC) [1]. CCl_4_ is a critical substance with a variety of uses including industrial manufacturing, household cleaners, degreasing, laundry and dry cleaning, fire extinguishers, refrigerant, and propellants, etc*.* However, CCl_4_ has many side effects on humans and the environment. It is therefore important to monitor, prevent and eliminate the harmful effect of CCl_4_ on the environment by environmental specialists, and on humans by considering thepathophysiology of side effects, clinical features, evaluation, and treatment of CCl_4_-induced toxicity [2, 3]. Human exposure to CCl_4_ takes place orally, dermally, or by inhalation [4]. The carcinogen exposure (CAREX) 1990–93 database from 15 European countries and the US National Occupational Exposure Survey 1981–1983 estimated potential exposure to CCl_4_ at around 70,000 people in Europe and 10,000 in the United States [2].

The production and use of CCl_4_, which can lead to the spread of CCl_4_ into the atmosphere, was largely controlled, except for special uses, after the Montreal Protocol (MP) 1987. However, CCl_4_ is still used in limited production processes for several hydrofluorocarbons (HFCs) and pyrethroid pesticides [5]. It is one of the main sources of groundwater pollution, that was very common in industrial complexes in the past [6, 7]. Previously, CCl_4_ was also used as a solvent in many applications. However, today it is widely used to create animal models of oxidative stress in laboratories [8]. CCL4 is one of the most commonly used agents to induce chronic and acute liver failure in experimental animals at doses of 0.5–2.5 mL/kg. Other pharmacological models of liver failure such as acetaminophen can be used, but rats are less sensitive to it`s toxic effects even at higher doses (300–1000 mg/kg IP) [9]. When CCl_4_ gets into the liver, it acts as a xenobiotic and converted into free radicals CCl_3_ and CCl_3_O_2_ by the P-450 monooxygenase-dependent microsome system and causes liver damage through membrane lipid peroxidation [10]. Exposure to CCl_4_ also causes free radical formation in many other organs, including the kidneys, testicles, lungs, and blood [11–13].

Herbal compounds derived from plant extracts that decrease chemically activating enzymes can be proper candidates for protection against toxicities caused by, for example, CCl_4_ and cisplatin as evidenced by inhibiting liver, kidney, brian damage in animal models [14]. Wild and cultivated mushrooms and several medicinal plants have been reported to have protective effects against CCl_4_-induced mostly liver, kidney and brain toxicity by modulating oxidative stress [15–17]. The Sophora plant belongs to the *Fabaceae* family. It has 52 species reported worldwide, with three species growing in Iran including *Sophora mollis*, *Sophora alopecuroides* and Sophora *pachycarpa (*S. *pachycarpa)*, each of which has subspecies. The branches grow from the stem of the plant. The leaves are composed, alternate, and pinnate with a length of 10–18 cm [18]. This plant is widespread in Southeast Europe, South Asia, Australia, the western Pacific, and South America [19]. In Iran, Sophora can be found countrywide, especially in arid and semi-arid regions [20, 21]. The roots of some species are widely used in traditional Chinese and Japanese medicine as antieczema, antidiarrheal, antipyretic, and anthelmintic agent [22]. Examination of the chemical composition of Sophora seed extract reveals the presence of quinolizidine alkaloids [23], prenyl flavonoids, and steroidal glycosides [24, 25]. Flavonoids have antiinflammatory [26, 27], immunomodulatory [28], and anticancer effects [29]. The presence of steroid flavonoids and glucosides as natural antioxidants in Sophora and its extract is believed to improve CCl_4_-induced oxidative stress and have beneficial effects on damaged organs. Therefore, in this investigation, the effect of pretreatment with hydroalcoholic extract obtained from *S. pachycarpa* seeds was examined against CCl_4_-induced acute toxicity on body organs, blood, and biochemical factors.

## Materials and methods

### Preparation of hydroalcoholic *S. pachycarpa* seed extract

The seeds of *S. pachycarpa* were purchased from the traditional market in Birjand, Iran. The plant material was identified and authenticated by a botanical specialist, Mr. Mohsen Pouyan (Medicinal Plants Research Complex, Academic Center for Education, Culture and Research, Birjand, Iran). A voucher specimen (voucher number: 9101) has been kept in our herbarium room for future reference. All plant protocols were performed in accordance with the Herbal Medicinal Product Committee (HMPC) (Ref. EMEA/HMPC/246816/2005) guideline and Ethic Committee of Birjand University of Medical Sciences Birjand, Iran. They were washed and dried in the shade and ground by a grinder. Next, their hydroethanolic extract was obtained by maceration method by mixing the powder and 80% ethanol in a ratio of 1:10 and placing it at 45 °C for 48 h with continuous stirring. The mixture was filtered using filter paper and concentrated under vacuum by a rotary evaporator (Heidolph, Schwabach, Germany). Finally,it was dried in a freeze dryer (Dena Vacuum Industry, model FD-5005-BT, Iran). The extract was stored at − 20 °C until used.

### Animal interventions

In this study, 40 male Wistar rats weighing 200–250 g were used. The rats were kept under stable physical conditions in an animal house at a temperature of 25 ± 2 °C and a light/dark cycle of 12 h. The rats had free access to standard food (Javane Khorasan Co.) and water and were grouped and caged 72 h prior to the study to adapt to the new conditions. Animal procedures were performed according to the ARRIVE guidelines [30]. All experimental procedures was approved by the Animal Ethics Committee of Birjand University of Medical Sciences, Birjand, Iran (IR.BUMS.REC.1399.126). We evaluated subacute pretreatment effects of *S. pachycarpa* for 21 days [31, 32]. The rats were then randomly divided into 5 groups (*n* = 8) [33, 34], including the Con group, which received normal saline (PO, 0.9%) for 21 days, the CCl_4_ group, which received normal saline (PO, 0.9%) for 21 days and CCl_4_ (IP, 1 ml/kg) on day 21, the Sp150 group, which received the hydroalcoholic extract of *S. pachycarpa* (PO, 150 mg/kg) for 21 days and CCl_4_ (IP, 1 ml/kg) on day 21, the Sp300 group, which received the hydroalcoholic extract of *S. pachycarpa* (PO, 300 mg/kg) for 21 days and CCl_4_ (IP, 1 ml/kg) on day 21, and the Sil group, which received silymarin (PO, 300 mg/kg) for 21 days and CCl_4_ (IP, 1 ml/kg) on day 21 [34–39]. Each doses of CCl_4_ was mixed with equal volume of olive oil as a vehicle and administrsted as a single IP dose at a doses of 1 ml/kg of rat body weight on day 21. The rats were weighted before treatment and then weekly to determine the exact amount of *S.pachycarpa* extract and CCL_4._

At the end of treatment, the rats were kept fasting for 12 h. They were then anesthetized with ketamine (65 mg/kg) and xylazine (10 mg/kg), and their blood was drawn via cardiac puncture. Some blood samples were collected in tubes containing EDTA and the remainder in test tubes, and their serum was then separated by centrifugation at 2500 rpm for 10 min and stored at − 20 °C to evaluate liver enzymes and biochemical factors.

### Biochemical evaluation

Serum levels of blood sugar, creatinine, urea, uric acid, and BUN in mg/dl, aspartate aminotransferase (AST), alanine aminotransferase (ALT), and alkaline phosphatase (ALP) in units per liter (u/l) and lipid profiles including serum levels of triglycerides (TG),total cholesterol, high-density lipoprotein (HDL), low-density lipoprotein (LDL), and very-low-density lipoprotein (VLDL) in mg/dl, using an appropriate biochemical kit according to instructions wereevaluated by an autoanalyzer (Roche Hitachi 912, Japan).

### Hematological evaluation

The levels of WBC, RBC, Hb, HCT, MCV, MCH, MCHC, PLT, neutrophils, monocytes, lymphocytes, and blood eosinophils were determined by a hematology analyzer (CBC Mindray BC-3000 Plus).

### Tissue samples preparation and liver and kidney antioxidant enzymes assay

Antioxidant enzymes were assayed in homogenized samples from the left liver (left lobe) and the left kidney. The samples were isolated and 500 μL of phosphate buffer (0.1 mol, pH = 7.4) was added per 100 mg of tissue and homogenized. The samples were then centrifuged at 4000 RPM for 4 min at 4 °C. To study the effects of *S. pachycarpa* seed extract and silymarin on enzyme levels in kidney and liver tissues, superoxide dismutase (SOD), catalase (CAT), and superfluid regenerative glutathione (GSH) were evaluated using standard kits (Navand Salamat Co., Iran. SOD catalogue number: NS-15033, CAT catalogue number: NS-15054, GSH catalogue number: NS-15087). The total antioxidant capacity (TAC) and peroxidation of kidney and liver tissue lipids were determined, respectively, by evaluating the regenerative ability of divalent iron (FRAP) and the level of malondialdehyde (MDA) marker using standard kits (Kavosh Ariyan Azma Co., Iran. FRAP catalogue number: Antox101, MDA catalogue number: Antox103).

### Histopathological evaluation

After the rats were anesthetized and their blood were drawn, samples of rat liver (right lobe), kidney, and testes were immediately isolated and placed in 10% formalin. For dehydration, they were placed in ethanol at concentrations of 50–100%. They were then clarified with xylol and placed in liquid paraffin at 60 °C. Sections of 4–5 μm were made from the samples and stained by hematoxylin–eosin (H&E) staining. Finally, 6 slides (9 parts each) were removed for microscopic examination by a blinded pathologist (Olympus Optical co., Tokyo, Japan).

### Statistical analysis

The data were reported as mean ± SD and analyzed using SPSS 22 software (SPSS Inc., Chicago, IL). For the normally distributed data, the ANOVA test was used to compare the differences between the groups, whereas the Kruskal-Wallis test with a significance level of *P* <0.05 was used otherwise. In addition, the Tukey and Mann-Whitney U post hoc tests were used to determine the differences between groups.

## Results

### Effect of *S. pachycarpa* seed extract on biochemical factors

The results of the serum levels of liver enzymes AST, ALT, and ALP showed that liver enzymes increased significantly in CCl_4_-poisoned rats compared to the controls (*P* < 0.05). The use of *S. pachycarpa* seed extract at a dose of 300 mg/kg prevented such an increase significantly (*P* < 0.05). Similar results were obtained using silymarin (*P* < 0.05) (Table 1).

**Table 1:** Effects of CCl_4_ and/or *S. pachycarpa* on serum biochemical parameters in rat

Parameters	Control	CCl_4_	Sp150	Sp300	Silymarin
SGOT (AST) (IU/L)	113.29 ± 17.52	545 ± 99.24***	488.67 ± 219.32	239 ± 33.71##	249.71 ± 143.77##
SGPT (ALT) (IU/L)	66 ± 12.97	646 ± 151.38***	456.33 ± 275.95	278 ± 42.03##	273.67 ± 167.74##
ALP (U/L)	232.57 ± 39.64	443.4 ± 193.92*	389.66 ± 67.21	254 ± 96.23#	320.85 ± 159.28
FBS (mg/dL)	138.14 ± 9.78	240 ± 66.52**	194.67 ± 17.95	184.67 ± 12.22#	156.43 ± 35.2##
Cholesterol (mg/dL)	124 ± 3	223.8 ± 23.34**	139.66 ± 15.14#	130.66 ± 8.32#	140 ± 38.94#
TG (mg/dL)	91.29 ± 14.2	104.8 ± 39.96	90 ± 47.28	103.66 ± 8.32	94.43 ± 25.57
HDL (mg/dL)	70.86 ± 13.01	65 ± 10.63	71.67 ± 7.37	60.33 ± 6.5	63 ± 10.29
LDL (mg/dL)	42 ± 11.91	79.4 ± 9.04***	48.67 ± 17.01##	42.33 ± 8.08###	46.43 ± 12.96###
VLDL (mg/dL)	18.14 ± 2.61	21.4 ± 6.87	18 ± 9.64	20.33 ± 2.3	16 ± 7.14
Creatinine (mg/dL)	0.86 ± 0.07	1.36 ± 0.28**	0.94 ± 0.23	0.95 ± 0.1#	0.91 ± 0.18#
Urea (mg/dL)	58.29 ± 12.27	83.6 ± 23.79*	75.67 ± 6.42	69.33 ± 6.65	71.14 ± 26.78
BUN (mg/dL)	27.22 ± 5.75	45.56 ± 13.37**	35.4 ± 8.31	35.36 ± 3	33.3 ± 1.46#
Uric Acid (mg/dL)	0.6 ± 0.21	2.32 ± 0.37***	1.11 ± 0.72#	1.31 ± 0.87	1.82 ± 0.56

The data in the table represent the mean ± SD (*n* = 8). * *P* < 0.05, ** *P* < 0.01 and *** *P* < 0.001 compared to control group. # *P* < 0.05, ## *P* < 0.01 and ### *P* < 0.001 indicate the difference between CCl_4_ group and other groups except the control group

Furthermore, the results of the mean serum level of blood sugar showed a significant increase in CCl_4_-poisoned rats compared to the controls (*P* < 0.05). The use of the *S. pachycarpa* seed extract at a dose of 300 mg/kg significantly inhibited such an increase (*P* < 0.05). Similar results were obtained using silymarin (*P* < 0.05).

Based on the results given in Table 1, the differences between the mean serum levels of lipid profiles TG, HDL, and VLDL were not significant in the groups (*P* > 0.05). However, there was a significant increase in the mean serum levels of LDL and total cholesterol in the CCl_4_-poisoned groups compared to the controls (*P* < 0.05). The dose-dependent use of *S. pachycarpa* seed extract expressed significantly reduced the serum levels of LDL and total cholesterol (*P* < 0.05), which was also the case in the Silymarin group (*P* < 0.05).

The mean serum levels of renal factors creatinine, urea, BUN, and uric acid are given in Table 1. CCl_4_ caused a significant increase in the serum level of renal factors in rats (*P* < 0.05). Similar to silymarin, the use of *S. pachycarpa* seed extract at a dose of 300 mg/kg significantly prevented the increase in the serum level of creatinine (*P* < 0.05). The mean serum level of uric acid was significantly reduced only in the group that received *S. pachycarpa* seed extract at a dose of 150 mg/kg (*P* < 0.05). Administration of the *S. pachycarpa* seed extract did not affect the serum level of urea and BUN.

### Effect of *S*. *pachycarpa* seed extract on hematological factors

The mean level of blood factors is given in Table 2. CCl_4_ did not cause any significant changes in the levels of WBC, HCT, MCHC, neutrophil, monocyte, lymphocyte, and eosinophil compared to the controls. However, it caused a significant decrease in the levels of RBC, Hb, MCV, MCH, and Plt compared to the controls (*P* < 0.05). The dose-dependent use of the *S. pachycarpa* seed extract significantly inhibited the reduction of RBC and Hb in the groups that received the extract compared to those that did not receive it (*P* < 0.05). Similar results were obtained with the use of silymarin (*P* < 0.05), which had the same effect in preventing the reduction of RBC as the effect of plant extract at a dose of 300 mg/kg. However, its effect on preventing the reduction of Hb was less than the effect of the plant on this parameter.

**Table 2:** Effects of CCl_4_ and/or *S. pachycarpa* on hematological parameters in rat

Parameters	Control	CCl_4_	Sp150	Sp300	Silymarin
WBC (1000/µL)	7.54 ± 2.37	10.16 ± 2.65	8.6 ± 0.52	7.33 ± 1.75	5.92 ± 0.86
RBC (Mil/µ)	8.31 ± 0.29	6.62 ± 0.6***	8.09 ± 0.61##	8.23 ± 0.55###	8.16 ± 0.31###
Hb (g/dL)	14.02 ± 0.38	12 ± 0.79***	13.86 ± 0.41##	14.13 ± 0.77###	13.4 ± 0.45#
HCT (%)	43.21 ± 1.29	41.18 ± 6.08	44.3 ± 1.22	43.8 ± 2.8	41.97 ± 1.88
MCV (fL)	52 ± 2.16	45 ± 5.46*	50.53 ± 4.14	51.86 ± 3.32	51.42 ± 1.48
MCH (pg)	16.87 ± 0.42	15.74 ± 0.85*	16.83 ± 0.7	16.83 ± 0.25	16.45 ± 0.23
MCHC (g/dL)	32.44 ± 0.73	29.58 ± 2.28	31.43 ± 4	31.8 ± 3.24	31.92 ± 0.6
Plt (1000/µL)	746.29 ± 251.13	392.2 ± 150.38*	680.67 ± 151.43	641.33 ± 126.95	762.5 ± 239.19
Neutrophils (%)	32.6 ± 11.54	48.84 ± 6.6	44.9 ± 8.19	36.6 ± 12.68	36.47 ± 15.36
Lymphocyte (%)	59.32 ± 8.89	50.52 ± 12.28	43.3 ± 5.11	39.33 ± 9.5	58.4 ± 17.25
Monocyte (%)	4.82 ± 2.58	6.92 ± 1.9	7.26 ± 1.8	7.43 ± 2.83	7.05 ± 1.16
Eosinophil (%)	3.24 ± 1.4	3.9 ± 1.83	4.53 ± 1.98	2.13 ± 1.81	3.25 ± 1.41

Data are means ± SD (*n* = 8). * *P* < 0.05, ** *P* < 0.01 and *** *P* < 0.001 compared to control group. # *P* < 0.05, ## *P* < 0.01 and ### *P* < 0.001 indicate the difference between CCl_4_ group and other groups except the control group

### Effect of *S. pachycarpa* seed extract on liver and kidney antioxidant markers

The results for hepatic and renal antioxidant markers are given in Table 3. According to the results, CCl_4_ caused a significant increase in the MDA (a marker of lipid peroxidation in the liver) compared to the controls (*P* < 0.05). The use of *S. pachycarpa* seed extract at a dose of 300 mg/kg significantly prevented lipid peroxidation (*P* < 0.05), while silymarin did not show such an effect. The MDA levels significantly decreased in the Sp300 group versus the Sp150 (*P* < 0.05).

**Table 3:** Effects of CCl_4_ and *S. pachycarpa* on antioxidant parameters in rat

Parameters	Tissue	Control	CCl_4_	Sp150	Sp300	Silymarin
MDA (µmol/L)	Liver	2.67 ± 0.56	7.85 ± 1.66***	7.72 ± 1.39	3.85 ± 1.49#$	6.5 ± 2.05
	Kidney	3.21 ± 1.22	4.81 ± 0.27*	4.31 ± 1.14	3.27 ± 0.58#	2.75 ± 1.06##
FRAP (µmol/L)	Liver	938.25 ± 129.54	343.4 ± 34.11**	394 ± 166.46	460.33 ± 124.26	555.75 ± 21.93#
	Kidney	1220.75 ± 162.5	594.25 ± 73.62*	1358.33 ± 153.08#	1218 ± 108.12#	1164.6 ± 34.68#
CAT (µg/g tissue)	Liver	2.75 ± 0.34	1.72 ± 0.37***	2.37 ± 0.39#	2.66 ± 0.2###	2.37 ± 0.42##
	Kidney	2.43 ± 0.53	1.49 ± 0.21***	1.48 ± 0.25	2.11 ± 0.23##$	2.34 ± 0.27###
GSH (µg/g tissue)	Liver	5.79 ± 2.15	3.3 ± 0.61**	5.1 ± 0.79	6.48 ± 0.78###	5.3 ± 0.67##
	Kidney	5.66 ± 0.85	2.16 ± 0.46***	2.91 ± 1.67	5.22 ± 0.85###$$	7.32 ± 1.23###
SOD (nmol/min/ml)	Liver	8.03 ± 1.1	5.95 ± 1.43*	7.24 ± 0.34	8.26 ± 1.86#	7.22 ± 1.72
	Kidney	9.04 ± 2.71	6.22 ± 1.51*	8.16 ± 2.16	8.5 ± 1.1	7.48 ± 1.04

The data in the table represent the mean ± SD (*n* = 8). * *P* < 0.05, ** *P* < 0.01 and *** *P* < 0.001 compared to control group. $ *P* < 0.05 and $$ *P* < 0.01 indicate the difference between Sp150 group and Sp300 group. # *P* < 0.05, ## *P* < 0.01 and ### *P* < 0.001 indicate the difference between CCl_4_ group and other groups except the control group

Comparison of the results for the FRAP (the marker of the total antioxidant capacity in the liver) also showed that CCl_4_ significantly reduced this marker compared to the controls (*P* < 0.05). Silymarin significantly inhibited the decrease in FRAP levels in the liver compared to the CCl_4_-poisoned group, while the *S. pachycarpa* seed extract did not show such an effect (*P* > 0.05). Evaluation of the liver tissue enzymes CAT, GSH and SOD showed that CCl_4_ significantly reduced the production of such enzymes compared to the controls, indicating damage to the liver (*P* < 0.05).

The dose-dependent use of *S. pachycarpa* seed extract significantly prevented damage to the liver tissue and thus prevented a decrease in CAT inthe groups that received the extract compared to control (*P* < 0.05). The use of the extract at a dose of 300 mg/kg also significantly prevented the reduction of liver SOD and GSH enzymes (*P* < 0.05), whereas the use of silymarin significantly prevented the reduction of CAT and GSH (*P* < 0.05) and had no significant effect on the SOD level ​​(*P* > 0.05).

The results for enzyme levels, total antioxidant capacity, and peroxidation of renal lipids (Table 3) showed that CCl_4_-induced toxicity caused significant changes in the marker levels in rats compared to the healthy controls (*P* < 0.05). Poisoning with CCl_4_ resulted in a significant increase in the level of MDA in rat tissues compared to the control group (*P* < 0.05). Such an increase indicates an increase in lipid peroxidation due to CCl_4_. The use of *S. pachycarpa* seed extract at a dose of 300 mg/kg, similar to silymarin, prevented lipid peroxidation. As a result, MDA levels were significantly reduced in the groups that received treatment (*P* < 0.05). According to Table 3, the total antioxidant capacity of kidney tissue measured via the FRAP method was significantly reduced in the CCl_4_-poisoned group compared to the healthy controls (*P* < 0.05). Administration of the *S. pachycarpa* seed extract at both 150 and 300 mg/kg doses or with silymarin significantly prevented the decrease in FRAP levels compared to the group that receive no treatment (*P* < 0.05).

CCl_4_ caused a significant increase in renal tissue enzymes CAT, GSH, and SOD compared to the healthy controls (*P* < 0.05). The use of either the *S. pachycarpa* seed extract at a dose of 300 mg/kg or silymarin significantly inhibited the decrease in the levels of CAT and GSH enzymes compared to the group that received no treatment (*P* < 0.05). *S. pachycarpa* seed extract at a dose of 300 mg/kg significantly increased CAT activity and GSH levels in the kidney of animals vesus the *S. pachycarpa* seed extract at a dose of 150 mg/kg (*P* < 0.05, *P* < 0.01, respectively).

In addition, receiving either silymarin or the *S. pachycarpa* seed extract did not significantly change SOD enzyme levels in the kidney compared to the group with no treatment (*P* > 0.05).

### Histopathology of liver, kidney, and testis tissues

**Fig. 1:**
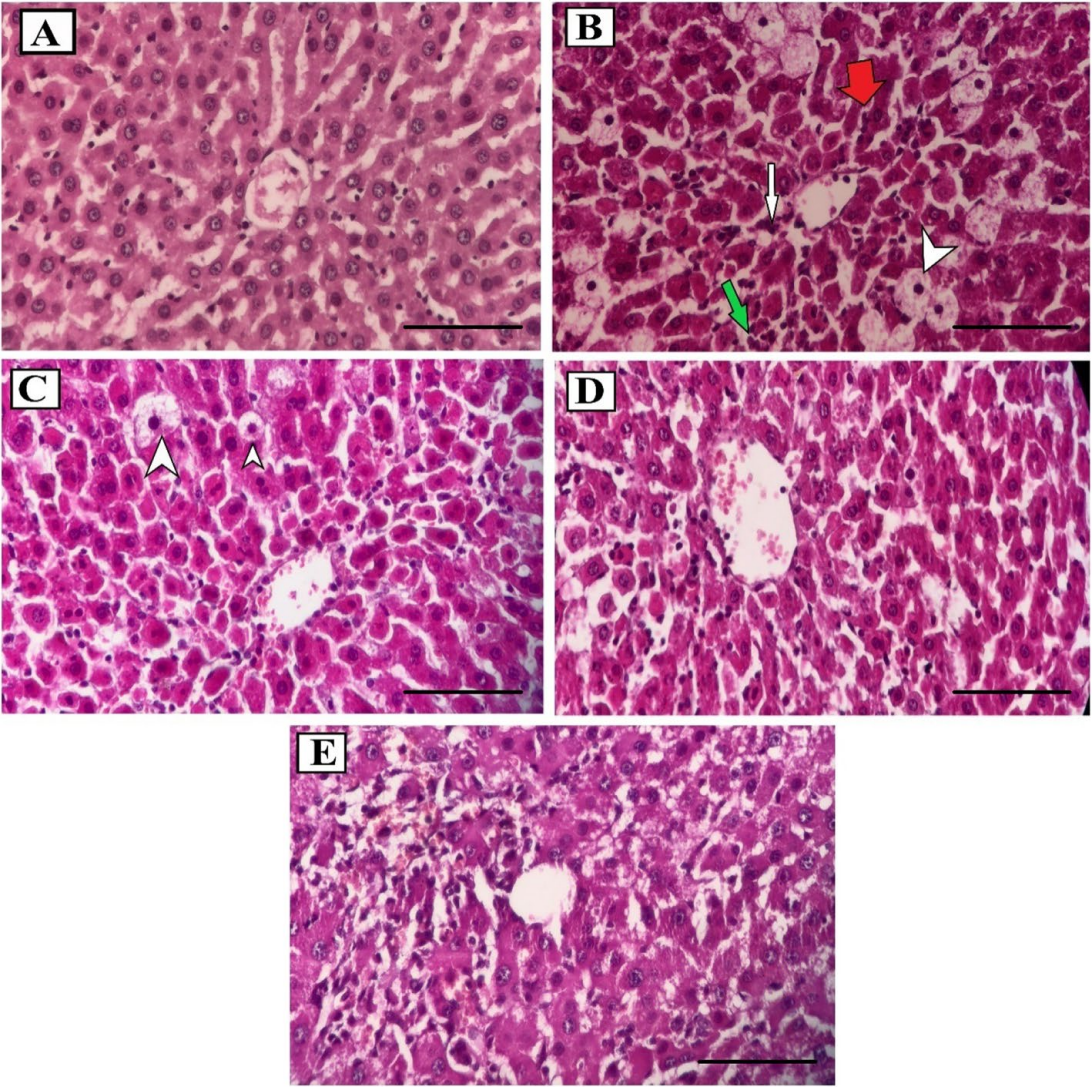
Histopathological effects of CCl_4_ and/or *S. pachycarpa* on the liver in rats. **A** Normal structure and architecture of liver tissue with (Control); **B** Sever centrilobular necrosis (white arrow), ballooning degeneration (white arrow tip), focal necrosis (green arrow), and inflammatory cell infiltration (red arrow) around of centrilobular vein (CCl_4_); **C** Moderate ballooning degeneration (white arrow tip) and focal necrosis (Sp150); **D** Normal structure and architecture of liver tissue (Sp300); **E** Normal structure and architecture of liver tissue with mild ballooning degeneration and inflammatory cell infiltration (Silymarin) (hematoxylin–eosin, × 400, Scale bar: 100 μm)

In order to confirm the findings of this study, pathological changes of liver, kidney, and testis were also studied. The centrilobular vein, around which blood flows with the lowest concentration of oxygen in the liver tissue, showed more severe cytotoxic changes in CCl_4_ exposure than in other parts of the tissue. Focal necrosis of hepatocytes (infiltration of neutrophils at the site of the hepatocyte) and ballooning degeneration and fatty change in the liver of the CCl_4_ group were significantly higher than the control (Fig. 1A) and other groups (Fig. 1B). In the use of *S. pachycarpa* extract at a dose of 150 mg/kg, centrilobular necrosis is less than the CCl_4_ group, as well as ballooning degeneration, fatty change, infiltration of inflammatory cells, and focal necrosis, although still seen sporadically, their severity is reduced (Fig. 1C). At a dose of 300 mg/kg, centrilobular necrosis, fatty change, and infiltration of inflammatory cells are greatly reduced and ballooning degeneration is almost not observed (Fig. 1D). In the silymarin group, there is also a significant decrease in pathological factors of liver tissue compared to the CCl_4_ group (Fig. 1E). Statistical results of histopathological changes in liver tissue show that these changes were significantly reduced by using * S. pachycarpa* at a dose of 300 mg/kg and even this extract was better than silymarin in improving the toxicity induced by CCl_4_. Table 4 shows in detail the scores assigned to each liver pathological change in different groups.

**Table 4:** Grading of the histopathological changes in liver tissues

Histopathological Changes	Control	CCl_4_	Sp150	Sp300	Silymarin
Centrilobular necrosis	0 ± 0	74.17 ± 9.97***	27.76 ± 4.28###	15.78 ± 6.54###	46.66 ± 9.49###
Ballooning degeneration	0 ± 0	79.16 ± 8.08***	47.23 ± 4.28###	5.55 ± 3.8###	15 ± 10.87###
Fatty change	0.83 ± 2.62	34.99 ± 11.64***	18.06 ± 6.27###	4.15 ± 3.5##	18.34 ± 6.98##
Inflammatory cell infiltration	2.49 ± 4	68.34 ± 6.58***	41.66 ± 7.46###	20.85 ± 4.54###	43.34 ± 6.98###
Focal necrosis	1.66 ± 3.49	67.5 ± 9.17***	29.15 ± 4.54###	11.1 ± 4.33###	45 ± 7.46###
Parenchymal architecture disruption	1.66 ± 3.49	68.33 ± 7.67***	38.88 ± 6.82###	19.45 ± 6.8###	36.66 ± 9.51###

The data in the table represent the mean ± SD (*n* = 8). * *P* < 0.05, ** *P* < 0.01 and *** *P* < 0.001 compared to control group. # *P* < 0.05, ## *P* < 0.01 and ### *P* < 0.001 indicate the difference between CCl_4_ group and other groups except the control group

**Fig. 2:**
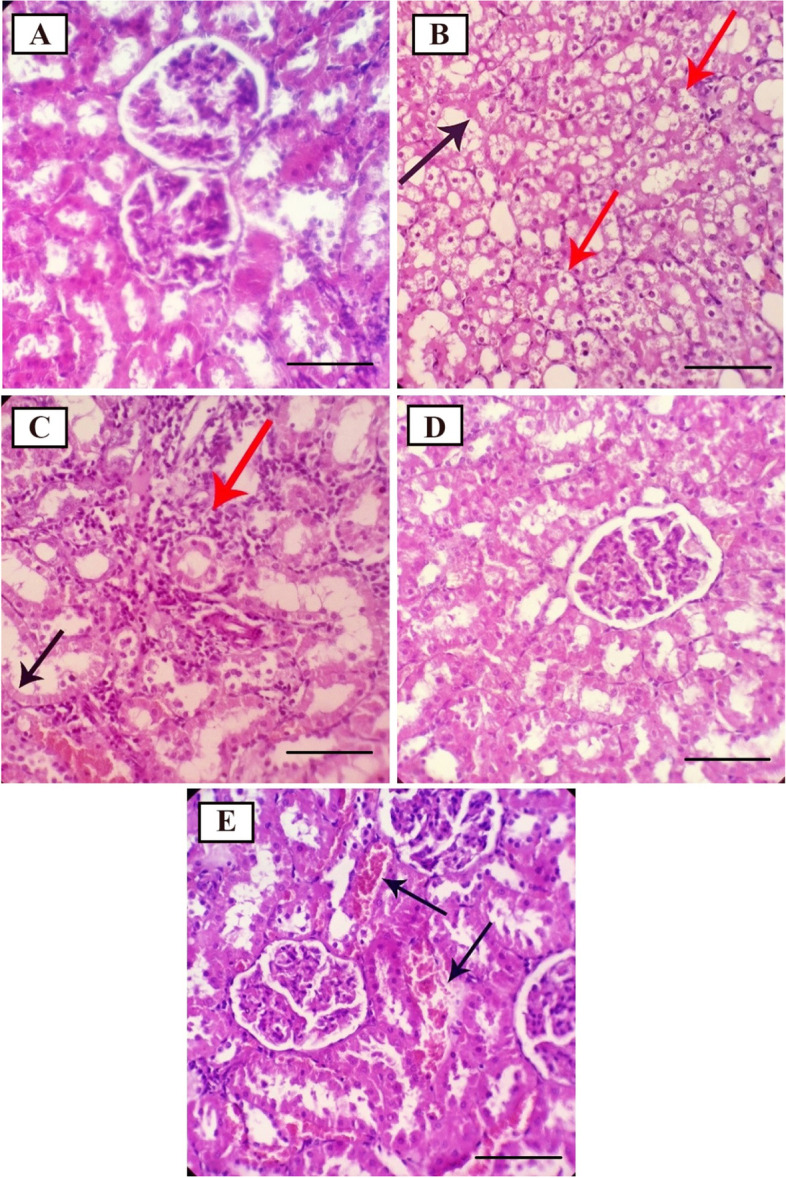
Histopathological effects of CCl_4_ and/or *S. pachycarpa* on Kidney in the rat. **A** Normal structure and architecture of renal tissue with intact glomeruli and renal tubules (Control); **B** Sever vacuolar degeneration (red arrows) and necrosis (black arrow) in renal tubules (CCl_4_); **C** Moderate necrosis in renal tubules (black arrow) associated with moderate interstitial mononuclear cell infiltration (red arrow) (Sp150); **D** Normal structure and architecture of renal tissue with intact glomeruli and renal tubules (Sp300); **E** Mild vascular congestion (arrows) (Silymarin) (hematoxylin–eosin, × 400, Scale bar: 100 μm)

As can be seen in Fig. 2, the use of tetrachloride in the group of CCl_4_ has caused severe vacuolar degeneration and necrosis in renal tubules (Fig. 2B). The use of *S. pachycarpa* seed extract prevented necrosis in renal tubules so that at dose 150 mg/kg the amount of necrosis was reduced and at dose 300 mg/kg necrosis expression was not observed (Fig. 2C and D). The use of silymarin also caused mild vascular congestion in kidney tissue (Fig. 2E).

**Fig. 3:**
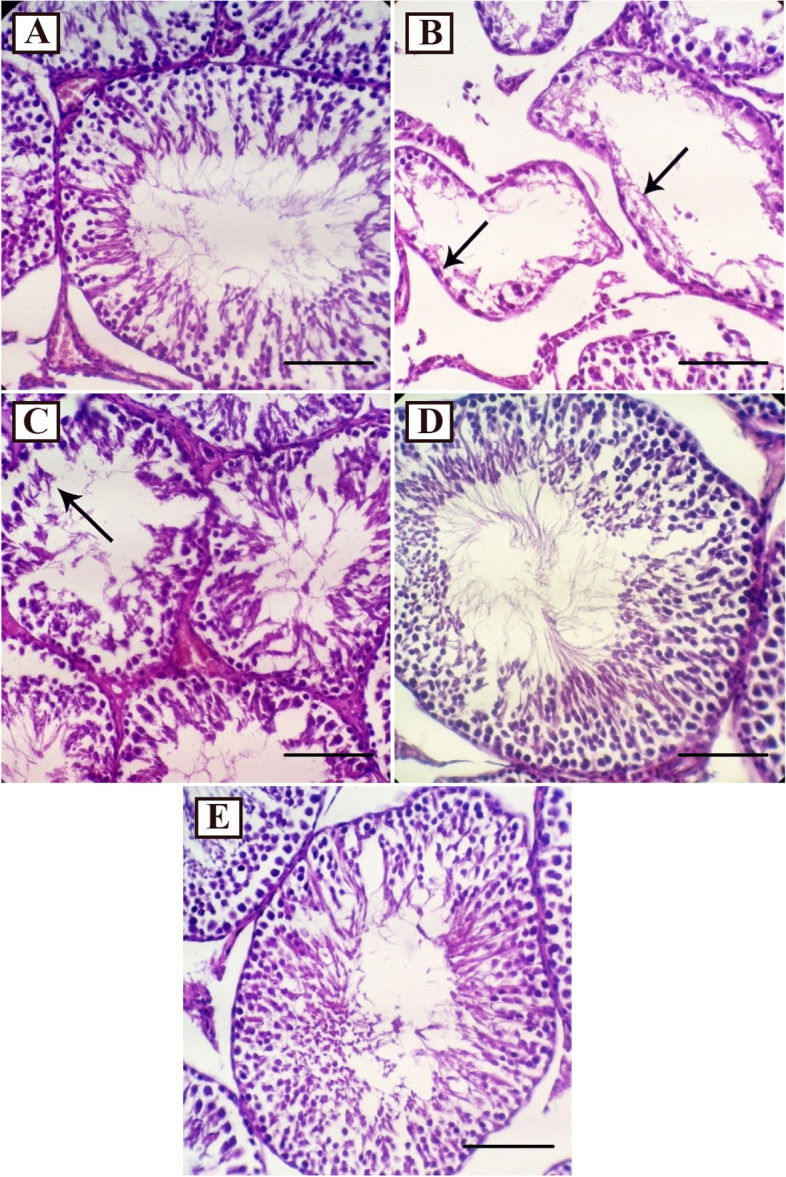
Histopathological effects of CCl_4_ and/or *S. pachycarpa* on Testis in rat. **A** The normal architecture of the seminiferous tubules with defined basement membrane and germinal layer (Control); **B** Sever vacuolation and degeneration of seminiferous epithelium associated with a reduction in germinal cells in the tubules (arrows) (CCl_4_); **C** Mild vacuolation and degeneration of seminiferous epithelium associated with reduction in germinal cells in the tubules (arrow) (Sp150); **D** Normal architecture of the seminiferous tubules with defined basement membrane and germinal layers (Sp300); **E** Normal architecture of the seminiferous tubules with defined basement membrane and germinal layer (Silymarin) (Hematoxylin and Eosin, × 400, Scale bar: 100 μm)

Pathological study of testicular tissue of the studied rats showed that CCl_4_ caused severe vacuolation and degeneration of seminiferous epithelium associated with a reduction in germinal cells in the tubules (Fig. 3B) compare to the control (Fig. 3A). The use of *S. pachycarpa* seed extract at a dose of 150 mg/kg reduced the toxic effects of CCl_4_ on testicular tissue by reducing vacuolation and degeneration of seminiferous epithelium associated with a reduction in germinal cells in the tubules (Fig. 3C). The use of *S. pachycarpa* seed extract at a dose of 300 mg/kg, as well as silymarin, similarly prevented the toxic effects of CCL_4_ on testicular tissue (Fig. 3D and E).

## Discussion

CCl_4_-induced organ damage is the best model of toxic agents-induced hyperglycemia, dyslipidemia, hepatotoxicity, reno toxicity, hepatotoxicity, and reproductive impairments and is usually used for evaluating the potential protective effects of medicinal herbs against organ toxicity [40]. The most important task of biochemical and histopathological assays is the identification the safety and effeciacy of *S. pachycarpa* against the CCl_4_ toxicity. The biochemical and histopathological tests is nessassary to determine the toxicity of the various compounds that are main indicators of organ damages [41]. Medicinal plants and their main ingredients have an important role in the inhibition of oxidative stress-induced by CCl_4_ in various tissues in experimental animals. The present study has focused on the potential antidote activity and possible mechanisms of action of *S. pachycarpa* seed extract in the prevention and management of hyperglycemia, dyslipidemia, hepatotoxicity, hematotoxicity, renotoxicity and testicular damage which were causedby acute exposure to CCl_4_ in male rats.

The close link between CCl_4_ exposure, insulin resistance, and hyperglycemia has been reported by several studies. Disruption of gluconeogenesis and glycogenolysis in the liver is triggered by CCl_4_, leading to hyperglycemia. Oxidative stress may be one of the molecular mechanisms underlying CCl_4_-induced hyperglycemia [42, 43]. The liver is the main target organ triggered by toxicants including CCl_4_ during their biotransformation [44]. Our data showed that CCl_4_ induced hyperglycemia accompanied by an elevation in liver enzymes including ALT, AST, and ALP. In addition, an increase in liver MDA levelswith a reduction in the FRAP and GSH levels as well as SOD and CAT activities can be assigned to the liver damage with CCl_4_ through stimulation of oxidative stress. Several histopathological damages including Sever centrilobular necrosis, ballooning degeneration, focal necrosis, and inflammatory cell infiltration around of centrilobular vein were observed in the liver of animals exposed to CCl_4_. Our findings confirmed the previous studies related to the hepatotoxicity of CCl_4_ [45–50]. It was proposed that the toxicity of CCl_4_ was related to the production of its reactive compounds including trichloromethyl (CCl_3_) and trichloromethylperoxy (CCl_3_OO) radicals [51]. The radicals induce lipid peroxidation in membranes and covalently bind to macromolecules in the hepatocytes, resulted in cell degeneration [52]. In the liver cells, CCl_4_ also disturbed hemostasis between lipid synthesis and degradation, resulting in dyslipidemia [53].Our results indicated elevated levels of cholesterol and LDL-C in the serum of CCl_4_-exposed rats.

We found that *S. pachycarpa* seed extract especially at high dose reversed the increase in FBS, serum liver enzymes activities, oxidative stress indices, and pathological damages in the liver as well as hyperlipidemia compared to the CCl_4_ group. The reason that can be mostly used to explain the hepatoprotective effects of *S. pachycarpa* seed extract is the presence of natural flavonoids such as polyphenolics with antioxidant properties [22, 54]. Our data confirmed studies indicated that the administration of *H. pedunculosum *and *O. basilicum* ameliorated the hepatotoxicity induced by CCl_4_ as evidenced by reversing increased liver enzymes and oxidative damage [55, 56]. This study was also similar to the findings of study conducted by Suzek et al., that indicated the ability of sweetgum oil, as a natural antioxidant, against hepatic damage induced by CCl_4_ via modulating oxidative stress [35]. It was suggested that *S. pachycarpa* seed extract contains important antioxidants which can neutralize ROS and decrease oxidative stress [57].

Inflammation is another important mechanism in CCl_4_-induced hepatotoxicity. Our histopathological findings showed infiltration of inflammatory cells in liver samples. We found that *S. pachycarpa* seed extract acted as an inflammatory inhibitor and provide partial protection against hepatotoxicity induced by CCl_4_ [58]. Due to the limitation of our study related to the evaluation of inflammatory molecular targets, we could not indicate the direct antiinflammatory effects of *S. pachycarpa*. Our study did not also show the significant effect of CCl_4_ on the WBC total and differential counts compared to the control group. Therefore, the antiinflammatory effects of this plan could not be explained exactly according to our findings. Controversially, previous studies indicated the ability of CCl_4_ to increase WBCs count through over-production of ROS in treated rats. This difference may be related to genetic background and individuals susceptible to the applied experimental animals in the present study that were resistant to alteration of leukocytosis induced by acute exposure to CCl_4_ [59, 60].

However, our data revealed that the decrease in RBCs count, Hb level, MCV, and MCH due to CCl_4_ administration could be related to the disruption in hematopoiesis and destruction of erythrocytes in the circulation. Additionally, the reduction of RBC and PLT counts may be associated with the inhibitory effect of CCl_4_ on erythropoiesis and thrombopoiesis in the bone marrow which is induced by oxidative stress. However, the administration of *S. pachycarpa* reversed these hematological changes to normal levels. Present data are in agreement with findings reported by Rahmouni et al*.* (2017) who demonstrated that administration of *Teucrium polium* aqueous extract prevented the hematotoxicity induced by CCl_4_ [61]. It was also confirmed findings of studies conducted by Doğan et al., (2018) that indicated the protective effects of *Agaricus arvensis* extract against erythrocyte fragility, hematological changes and oxidative stress induced by CCl_4_ in rats [62, 63]. The amelioration effects of *S. pachycarpa* on hematological parameters could be related to the presence of main ingredients with antioxidant activities in this plant [64].

Similar to the previous findings, we found that CCl_4_ caused nephrotoxicity directly via free radical production [51, 65]. Our data was also confirmed that antioxidant therapy could inhibit CCI_4_-induced kidny damage as evidenced by ameliorating biochemical and histological changes in rats [66]. Our study also augments the protective activity of *S. pachycarpa* against oxidative stress induced by CCl_4_ in renal tissue. We found that this plant extract, especially at a higher dose, decreased creatinine and BUN accompanied bya decrease in kidney MDA levels as well as an increase in GSH and FRAP levels and also CAT and SOD activities. Renohistology of CCl_4_ exposed animals revealed severe vacuolar degeneration and necrosis in renal tubules, which was significantly ameliorated by *S. pachycarpa* administration. The present finding was in agreement with earlier findings related to the protective effects *Monotheca buxifolia* against CCl_4_-stimulated renotoxicity in rats [67].

It was also found that elevation in CCl_4_-induced oxidative stress in testicular tissue was associated with infertility in male rats. We found that CCl_4_ induced severe vacuolation and degeneration of seminiferous epithelium associated with a reduction in germinal cells in the tubules. Our histopathological findings were in accord with the results of the study conducted by Sahreen et al. (2013) which indicated degenerative damages including loss of germ cells, germinative epithelium disruption, interruption in meiosis, abnormal shape of sperm, and seminiferous tubules delocalization. Similar to* S. pachycarpa*, i﻿t wa﻿s found that *Rumex hastatus* repaired testicular tissue and sperm abnormalities [68].

Enzymatic and non-enzymatic antioxidants are an important part of the mechanism for preventing oxidative damage [69]. In this study, a decrease in the antioxidant content in the liver and kidney following CCl_4_ administration, which may be related to oxidative modification of antioxidant content and inactivation of the enzyme upon ROS overproduction. These findings were in agreement with previous studies [70, 71]. *S. pachycarpa* extract increased antioxidant content of the liver of kidney versus the CCl_4_-exposed group. These data confirm previous results about the antioxidative activity of *S. pachycarpa* in various tissues [34, 54, 72]. *S. pachycarpa* has been found as an antioxidant [54], and is involved in the direct neutralization of free radicals, mediated by flavonoids present in *S. pachycarpa*.

In conclusion, the data obtained in our study confirm the protective effect of *S. pachycarpa* against acute exposure to CCl_4_-induced organ toxicity in rats, which is evidenced by improvement in hyperglycemia, dyslipidemia, hematotoxicity, hepatotoxicity, renotoxicity, and testicular damage. More studies are required to characterize the active ingredients of *S. pachycarpa* extract and to find molecular mechanisms underlying its protective effects against CCl_4_.

## Supplementary Information

**Additional file 1. **

## Data Availability

The data supporting the findings of this study are available within the article and its supplementary materials.
